# Cryo-electron Microscopy Analysis of Structurally Heterogeneous Macromolecular Complexes

**DOI:** 10.1016/j.csbj.2016.10.002

**Published:** 2016-10-14

**Authors:** Slavica Jonić

**Affiliations:** IMPMC, Sorbonne Universités - CNRS UMR 7590, UPMC Univ Paris 6, MNHN, IRD UMR 206, 75005, Paris, France

**Keywords:** Cryo-electron microscopy, Single particle analysis, Macromolecular complexes, Heterogeneity, Structure, Flexibility, Conformational changes, Dynamics

## Abstract

Cryo-electron microscopy (cryo-EM) has for a long time been a technique of choice for determining structure of large and flexible macromolecular complexes that were difficult to study by other experimental techniques such as X-ray crystallography or nuclear magnetic resonance. However, a fast development of instruments and software for cryo-EM in the last decade has allowed that a large range of complexes can be studied by cryo-EM, and that their structures can be obtained at near-atomic resolution, including the structures of small complexes (e.g., membrane proteins) whose size was earlier an obstacle to cryo-EM. Image analysis to identify multiple coexisting structures in the same specimen (multiconformation reconstruction) is now routinely done both to solve structures at near-atomic resolution and to study conformational dynamics. Methods for multiconformation reconstruction and latest examples of their applications are the focus of this review.

## 1 Introduction

Recent instrumental and methodological developments for cryo-electron microscopy (cryo-EM) [1], [2], [3], [4], [5], [6], [7], [8], [9], [10], [11], [12], [13], [14], [15], [16], [17], [18], [19] made that the structures of macromolecular complexes are now often determined at subnanometer and near-atomic resolutions [20], [21], [22], [23], [24], [25], [26], [27], [28], [29], [30], [31], [32], [33], [34], [35], [36], [37], [38], [39], [40], [41]. The most exciting results in terms of resolution and size of solved structures are currently being obtained with the latest-generation cryo-electron microscopes equipped with direct electron detectors (registering electrons directly rather than via a scintillator and recording movies allowing for correction of the specimen motion occurring during beam exposure) and software for automated collection of images, in combination with the use of advanced image analysis methods and high performance computing platforms [42], [43], [44].

First structures at near-atomic resolution were obtained for large complexes with high symmetry such as icosahedral-symmetry viruses [20], [21]. However, several works have recently shown that cryo-EM can be used for near-atomic resolution of structures of small complexes (170–500 kDa) with low symmetry [22], [27], [41] or no symmetry [26], [32], where the best resolution (1.8 Å) was obtained for 334 kDa glutamate dehydrogenase [40]. Bartesaghi and collaborators have pointed out that, rather than imaging technologies or image-processing methods, the major bottleneck to a routine cryo-EM determination of structures at resolutions close to 2 Å is currently the preparation of specimens of adequate quality that takes into account intrinsic protein flexibility [27]. Regarding larger complexes, subnanometer resolution is currently often achieved [24], [25], [28], [30], [36] and near-atomic resolution is becoming more and more frequent [23], [29], [31], [33], [34], [35], [37], [38], [39].

Three-dimensional (3D) reconstruction from heterogeneous sets of images normally results in low-resolution density maps. Thus, data heterogeneity analysis to isolate images of complexes of similar molecular compositions and conformations is a usual prerequisite to structural determination at high resolution. Biochemical procedures can usually be optimized so that the majority of complexes in the specimen, if not all of them, have the same molecular composition. However, the same composition rarely means the same conformation, due to the flexibility of complexes. Thus, conformational heterogeneity of specimens is usually analyzed by image analysis and classification methods. The reconstruction of different coexisting structures from the same sample will here be referred to as multiconformation reconstruction. It involves a classification strategy that assigns the particles having similar structures (similar molecular compositions and similar conformations) to the same class of particles. Multiconformation reconstruction is used to obtain high-resolution structures and provides insights into conformational dynamics of macromolecular complexes. Multiconformation reconstruction methods will be reviewed here together with the latest examples of their applications.

**Fig. 1:**
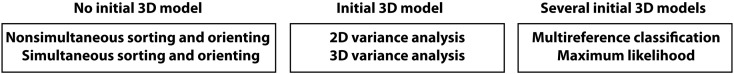
Multiconformation reconstruction methods reviewed here.

Image classification in multiconformation reconstruction can be supervised or unsupervised. Supervised classification aims at sorting images into classes based on information on expected conformational states (prior knowledge about the distribution of states), and its use is limited to studying systems where this prior information is available. The majority of methods reviewed here belong to the group of unsupervised classification methods whose aim is to find actual conformational states without a prior knowledge about the distribution of states. Due to computational limitations, the majority of available multiconformation reconstruction methods assume specimens with relatively few different conformations of complexes (usually, less than 10) and restrained compositional heterogeneity. They also take into account that biochemical preparation of specimens is usually optimized to reduce the number of different structures coexisting in the same specimen. These methods are sometimes referred to as discrete conformational heterogeneity methods. They differ in the number of required initial 3D models (0, 1, or several) (Fig. 1), but a more important difference among these methods is whether they analyze heterogeneity at image level (in a 2D space) or at volume level (in a 3D space). Thus, these methods will here be grouped in two groups (2D and 3D heterogeneity analysis methods) and reviewed in two separate sections.

Development of methods for analyzing quasicontinuums of conformational states is an active field of research that will here be only briefly discussed (Outlook section). These methods will be fully reviewed in a separate publication.

## 2 2D Heterogeneity Analysis Methods

In this section are reviewed methods that perform 3D reconstruction of different structures identified by analyzing structural heterogeneity at the level of images. Some of these methods use 3D starting models to determine the orientation of images while the other methods, referred to as ab initio methods, use no prior structural information.

### 2.1 Orientation Determination Without a Starting 3D Model

The orientation of images can be determined based on the central section theorem [45]. This theorem states that the Fourier transform of a 2D projection is a plane intersecting the origin of the 3D object's Fourier transform and that this plane is parallel to the projection plane [45], [46]. Any two non-parallel 2D projections of the same 3D object will therefore share a common line in Fourier space. Thus, the orientation of images can be determined by determining the relative orientation of common lines between the 2D Fourier transforms of images [47], [48]. The 3D model of the object obtained using images and the determined orientation is referred to as ab initio 3D model.

If the given set of images is heterogeneous, the images have to be sorted into structurally homogeneous subsets (image sorting) and 3D geometrical relationships among the images have to be determined (image orienting). When using no prior 3D model, image sorting and orienting can be performed in two separate steps or simultaneously. In the two-step approach proposed in [49], image orienting is preceded by a classification of images in classes of similar orientations (orientation classes) and a classification of each orientation class in classes of similar structures (image sorting), and both classifications are based on 2D multivariate statistical analysis (MSA) [50], [51]. This approach, here referred to as nonsimultaneous sorting and orienting, has been particularly efficient in separating small and large particle images or images of ligand bound and unbound complexes [49], [52], [53]. In the approach for simultaneous sorting and orienting proposed in [54], all 6 parameters (3 Euler angles, 2 shifts, and structure assignment) are considered simultaneously for all images by solving a multidimensional optimization problem and common line correlations in Fourier space [54]. The larger the expected number of different structures, the more complex is the optimization problem to solve. So far, this approach was only used to separate two conformational states, such as open/closed and ligand bound/unbound states [54], [55].

The main problem with the methods in this group is their low robustness to noise. They are thus usually used with 2D average images that have a higher signal-to-noise ratio (SNR) than individual images [53], [55]. Also, their applications in studies with more than two conformational states have not yet been demonstrated.

### 2.2 Orientation Determination Using a Starting 3D Model

Methods in this group aim at facilitating recognition of structural variability by minimizing orientational variability. They assume that dissimilarities between images corresponding to different structures are larger than those between images corresponding to the same structure but having slightly different angular directions.

The orientational variability is minimized by determining the orientation of images with respect to a preliminary 3D model that is usually obtained by combining images from the entire heterogeneous data set. Images assigned to the same projection direction are then sorted in clusters by analyzing discrepancies between common lines [56], [57], [58] or between entire images or their regions [59], [60], [61], [62]. Clusters in each projection direction are labeled (different structures are assigned to different clusters) and those with the same label in different projection directions are combined in the same 3D reconstruction. Cluster labeling is a difficult task and the labeling approaches are usually not trivial. For instance, in [57], distinct cluster averages corresponding to a selected view (the view selected visually as the view showing the highest variability) and presumably representing different conformers are used as conformational references for the conformational assignment of cluster averages in all other orientations based on the highest cross-correlation of common lines between the cluster averages and the conformational references. On the contrary, the approach proposed in [58] considers all cluster averages simultaneously instead of selecting a single representative view and defining conformational references, by computing all pairwise similarities between the cluster averages based on cross-correlation of common lines.

The preliminary 3D model should have good quality and a potential model bias should be considered. In Fig. 1, these methods are referred to as 2D variance analysis methods to be distinguished from the 3D variance analysis methods that also use an initial 3D model to orient images but analyze heterogeneity at volume level (classification based on 3D variance analysis that is described below).

The 2D heterogeneity analysis methods have also been used with globally homogenous data sets to select the most self-consistent subset of particles for a high-resolution 3D reconstruction. For instance, a procedure involving MSA-based classification of images, ab initio reconstruction, and iterative refinement has recently resulted in the first subnanometer-resolution structures of the complete portal-phage tail interface that mimic the states before and after DNA release during phage infection [63].

## 3 3D Heterogeneity Analysis Methods

In this section are reviewed methods that perform 3D reconstruction of different structures identified by analyzing heterogeneity at the level of 3D reconstructions. These methods require one or several initial 3D models and can be classified in the following three groups: multireference classification, classification based on 3D variance analysis, and classification based on maximum likelihood.

### 3.1 Multireference Classification

Methods that sort images by evaluating their similarity with a set of structures manually selected to contain expected features (3D references) [64], [65] belong to the class of supervised classification methods and will here be referred to as supervised multireference classification methods. In these methods, all given 3D references are first projected in directions uniformly distributed over a 3D sphere. Then, each particle image is compared with all obtained 2D projections (via projection matching that involves rotational and translational alignment of images with projections and computing their cross-correlation coefficients) to find the most similar projection and to assign the particle image to the corresponding 3D reference (the 3D reference from which the most similar 2D projection comes from). Each obtained class thus contains images that are the most similar to one of the given 3D references. Finally, a 3D reconstruction is computed from each obtained class of images using the rotational and translational parameters determined via the alignment with the best-matching reference. The required important prior knowledge (already available 3D template structures for all conformational states potentially present in the sample) makes supervised multireference classification methods useless when this prior information is not available, which is often the case.

Another group of multireference classification methods “select 3D references” automatically, via an iterative procedure in which 3D references compete for particles and are updated in each iteration [66], [67], [68], [69], [70]. Such methods belong to the class of unsupervised classification methods and they will here be referred to as unsupervised multireference classification methods. As supervised multireference classification methods, unsupervised multireference classification methods first assign each image to the best-matching reference from the set of given 3D references (by projection matching) and then compute 3D reconstruction from images assigned to the same 3D reference. However, contrary to supervised multireference classification methods, these methods use the 3D reconstructions obtained in the first iteration to update the 3D references for the next iteration. The iterations consisting of alignment, classification, and reconstruction steps are repeated until obtaining stable 3D references, which allows obtaining new structural features (on the 3D references) with new iterations. An iterative procedure consisting of alignment-classification rounds was initially used in 2D work (using 2D class averaging instead of 3D reconstruction from image classes), where it was referred to as multi-reference alignment [71]. This approach is a version of K-means clustering algorithm, which estimates the unknown cluster centers based on the data and assigns the data to the nearest cluster (the nearest cluster center). In the EM context, the cluster centers are the reference structures (at a current iteration) and the usual measure of distance between images and the centers of clusters is the correlation between the images and the projections of the reference structures. Another version of this approach adds new 3D references progressively [72]. This approach, referred to as incremental K-means-like approach [72], starts by aligning the entire set of images using only one initial reference. After stabilizing the particle orientation parameters, it adds a new reference; if the two references are not too similar, the second reference will attract the particles that do not fit well the first reference and these particles will be used to update this second reference. After a refinement of the two-reference alignment (using a decreasing angular step size and a decreasing search range) and a stabilization of the orientation parameters, a new reference may be added and the process repeated until the number of references starts to exceed the number of intrinsic divisions within the dataset, which can be observed as a poor reconstruction (usually from a very small subpopulation of particles) after adding a new reference [72]. The particle images assigned to corrupt reconstructions are assumed to contain degraded particles and are removed [72]. The incremental K-means-like approach was recently used for isolating rotated from unrotated 80S ribosome images (using a low-resolution 80S ribosome from rabbit as reference) before their further splitting guided by 3D variability analysis (see the next subsection), and this combined procedure resulted in solving 11 states with conformational and compositional differences along the elongation circle of human 80S ribosome [73].

The main problem with multireference classification methods is a potential bias with the provided initial 3D references, which is usually observed as absence of new structural details on the reconstructed density maps. The initial references could be obtained by angular reconstitution, but they should be correct enough to avoid incorrect classifications. Also, a higher number of required references results in a lower performance of these methods.

These methods can also be used to separate particles to extract the most self-consistent data subset for a high-resolution 3D reconstruction. For instance, a near-atomic resolution of Porcine circovirus PCV2 virus-like particle was recently obtained using a strategy based on multireference alignment, classification, and refinement [39].

### 3.2 Classification Based on 3D Variance Analysis

Methods in this group are based on the principle of estimating a global 3D variability from the variability of data subsets. They require a prior knowledge of 3 Euler angles and 2 in-plane shifts for each particle image. However, they do not require that the number of different structures (classes) is known a priori. Instead, the number of classes can be determined a posteriori (after the 3D variability analysis) so that the classes show high inter-class and low intra-class variances.

One approach is to compute the 3D variance volume using a series of 3D reconstructions from bootstrap random sampled subsets of a given set of images. Bootstrap resampling was introduced to EM by Penczek and collaborators who proposed to generate new sets of images in which some images randomly selected from the given data set are duplicated and some are omitted so that the new data sets have the same size as the given data set [74]. The global 3D variance is then computed using a series of 3D reconstructions obtained from the new data sets [74]. In [75], this 3D variance was used for localizing variable areas in a preliminary 3D model (obtained by combining the entire heterogeneous data set) and sorting particles in two classes corresponding to ligand binding and unbinding. More precisely, a 3D spherical mask placed in the region of highest 3D variance was projected in different directions to create 2D binary masks, and images with higher average densities in the regions outlined by respective 2D masks were labeled as ligand-bound states while those with lower average densities were labeled as ligand-unbound states [75]. Classification in a localized (masked) region is known as focused classification [75]. In another approach, the 3D variance was obtained by backprojection of 2D variance images calculated from images with the same projection directions and it was used in a focused classification approach to separate ligand-bound states from ligand-unbound states along the elongation circle of human 80S ribosome [73]. Also, classification approaches based on eigen analysis (multivariate statistical analysis or principal component analysis) of a series of 3D reconstructions from random subsets of images have been proposed [68], [76], [77] as well as approaches to estimate the actual covariance matrix or its eigenvectors directly from images [78], [79], [80], [81].

### 3.3 Classification Based on Maximum Likelihood

Maximum-likelihood (ML) estimation was introduced to EM by Provencher and Vogel in the context of 3D reconstruction of viruses and ribosomes [82], [83]. Later, an improved ML-based virus reconstruction approach was developed by Doerschuk and Johnson [84] and a 2D ML approach to general single-particle structure determination was proposed by Sigworth [85]. An extension of this latter approach to 3D, involving a membership of each experimental image to one of a fixed number of classes, was proposed by Scheres and colleagues, and it is referred to as ML3D [86]. ML3D considers that image orientation and class membership are random variables and computes 3D models by expectation–maximization (E-M) recursive optimization. E-M algorithm is equivalent to a multireference projection-matching algorithm, where the discrete assignments of image orientation, translation, and class membership are replaced with probability-weighted integrations over all possible assignments and each image contributes to all orientations, translations, and classes with some weight. In practice, integrations are replaced with summations over a set of discrete projection directions, in-plane rotations, in-plane translations, and classes. 3D ML-based cryo-EM image classification was first demonstrated using ML3D, which resulted in solving structures of 70S ribosomes with bound and unbound elongation factor G that were consistent with those obtained by supervised multireference classification [86].

ML3D is available in XMIPP [10], [87], [88] and it is usually limited to intermediate resolution analysis because refinements with angular sampling steps finer than 10° are difficult to perform in practice [89]. The main reason for this is that ML3D keeps reference projections in computer memory to speed up computations, which makes that memory use becomes too large for angular sampling steps finer than 10° [86], [89]. The two most recent and currently most popular ML-based methods overcome this problem by computing reference projections on-the-fly instead of precomputing and storing them in computer memory, thanks to the use of central section theorem for the projection operation. One of these two methods is available in RELION [6], [90] while the other is available in FREALIGN [12], [91]. Their performance is similar, though the implementation in RELION may give slightly better results in some data cases [12]. Both of them estimate parameters of prior distributions from data using variants of ML methodology and are thus referred to as extended likelihood or empirical Bayes [12].

To allow efficient optimization, ML-based methods simplify mathematical expressions by assuming independence of pixels and Gaussian white noise in either real space or reciprocal space. The approach assuming independence of real-space pixels cannot allow for correlations among real-space pixels that are due to the contrast transfer function of the electron microscope, while those assuming independence of reciprocal-space pixels cannot allow for real-space masking of noise around the particle to improve the SNR as real-space masking introduces correlations among reciprocal-space pixels [12], [90]. ML3D and the FREALIGN approach assume independence of real-space pixels while the RELION approach assumes independence of reciprocal-space pixels. The zero-mean Gaussian prior on the reciprocal-space pixels in RELION makes a smoothing effect that reduces noise in the reconstruction. One can also note that the ML-based approach of RELION is an improved version of the Fourier-space version of ML3D that is referred to as MLF3D [92] and available in XMIPP (MLF3D performs many operations in the same way as ML3D and the computational costs of MLF3D and ML3D were observed to be comparable [92]). FREALIGN performs ML classification using E-M algorithm with fixed alignment parameters found by a non-ML parameter refinement. Rounds of non-ML parameter refinement are typically alternated with rounds of the E-M algorithm, which makes the method more efficient than implementations that are entirely based on ML [12].

ML-based methods can be used with heterogeneous data in order to determine multiple co-existing conformational states, but they can also be used with globally homogenous data, in which case the most self-consistent subset of particles could be isolated to obtain a single-state reconstruction at high resolution. A few examples of these two types of applications of RELION and FREALIGN ML-based methods will be cited here. It should however be noted that the initial models used by these approaches are usually obtained by other approaches (e.g., initial model generation methods available in EMAN2 and SIMPLE software packages [3], [18], random conical tilt technique [93], or selecting models from EMDB), which was also the case in the applications that are cited next.

In the context of identifying the most self-consistent subsets of particles for reconstructing single states at high resolution, the RELION approach resulted in near-atomic resolution of different structures, from small complexes, such as a 170 kDa asymmetric gamma-secretase [26] and a 300 kDa tetrameric TRPV1 ion channel [22], to large complexes, such as a 4.3 MDa asymmetric 80S ribosome [31] and a 5.5 MDa tetrameric ryanodine receptor RyR1 [35]. In the same context, the FREALIGN approach resulted in near-atomic resolution of structures of complexes such as a 250 kDa asymmetric L-protein of vesicular stomatitis virus [32], a 465 kDa tetrameric beta-galactosidase [27], and a 540 kDa hexameric AAA + ATPase p97 [37].

In the context of classifying heterogeneous data sets to identify and structurally characterize different conformational states that coexist in the specimen, the RELION approach was combined with the use of masks and a modification of experimental images to improve consistency of their comparison with model projections. This approach separates experimental images based on their differences in a region of interest, which involves subtracting a residual signal (the signal outside the region of interest, but inside the complex) from the experimental images. The modification of experimental images improves consistency of their comparison with model projections in the region of interest, but it requires a preliminary determination of image orientation and translation parameters and a creation of two 3D masks, one for the region of interest and the other for the entire complex. This strategy produced results such as three distinct conformations of human gamma-secretase [94] and different local-region conformations of the yeast spliceosomal U4/U6.U5 tri-snRNP [95]. Regarding characterization of coexisting conformational states with FREALIGN, several examples of results have been recently obtained, such as three distinct coexisting functional states of human p97 with occupancies of zero, one, or two molecules of ATPγS per protomer [37], seven distinct states of bovine mitochondrial ATP synthase with different modes of bending and twisting [96], and five 80S ribosome structures formed with the Taura syndrome virus internal ribosome entry site (IRES) and eEF2-GTP-sordarin suggesting a trajectory of IRES translocation required for translation initiation [97].

The main problem with ML-based classification methods is that the number of classes should be known from the beginning. To avoid using arbitrary numbers of classes, it is recommended to test different numbers in order to find the largest number that still produces classes with different features [89]. Also, there is a risk of bias to initial models, and to reduce it, the initial models are usually low-pass filtered to 40–80 Å.

## 4 Outlook

With specimens containing a small number of different conformations of the same complex, methods reviewed in this article can lead to near-atomic resolution of the different conformations. However, it would be difficult to use such methods with specimens containing a quasicontinuum of conformational states (a large number of discrete samples from a continuous trajectory of conformational change), as a meaningful definition of a few discrete classes would be impossible in this case. Several methods have been recently developed that could help analyzing continuous conformational changes [79], [81], [98], [99], [100]. However, only two of them explicitly consider continuous conformational changes while aiming at visualizing the entire conformational distribution, animating trajectories of conformational changes, and reconstructing 3D structures along these trajectories. One of them is based on normal mode analysis [99], [101] while the other is based on manifold embedding [98], [102]. The problem of quasicontinuums of conformational states is challenging and it is not yet fully solved. Development of methods for their study is the first step towards full studies of dynamics of macromolecular complexes. Instead of biochemically isolating a few conformational states, the concept of studying entire macromolecular dynamics is based on the principle of allowing as many as possible different conformational states in the specimen. Because of the expected high impact, methodological developments for such studies are currently in progress and will be reviewed in a separate publication.

## 5 Summary

In this article, I reviewed methods for analyzing structural heterogeneity of macromolecular complexes in cryo-EM images and examples of recent applications of these methods. These methods assume specimens containing a small number of different coexisting structures (typically, less than 10) and differ in the way of analyzing the heterogeneity (2D or 3D analysis) and in the number of required initial 3D models (0, 1, or several). When specimens contain complexes with a small number of different structures, 3D reconstructions at near-atomic resolution may result from the classes of images obtained with these methods, as shown in several works cited in this review. However, their use is limited in the case of continuous conformational changes because of potentially large numbers of different conformational states that could be collected in this case (the large number of states would be difficult to classify in a relatively small number of classes used by these methods).

Except for supervised multireference classification methods, all methods reviewed here belong to the class of unsupervised classification methods, which aim at finding actual conformational states without a prior knowledge about the distribution of states. Methods that do not use available 3D models (ab initio methods) have advantage over other methods regarding the model bias, but they base the 3D reconstruction of different conformations on conformational variability analysis and sorting in 2D space. Methods that attack the heterogeneity problem directly in 3D space are more straightforward, but as they use available 3D models (at least for initial image orientation), the potential model bias should be carefully considered when using these methods. Currently, the most popular 3D methods are maximum-likelihood-based classification methods available in RELION [6], [90] and FREALIGN [12], [91] software packages.

This article is a quick start overview of cryo-EM image analysis methods that deal with discrete conformational heterogeneity of macromolecular complexes. In this context, the most relevant and recent articles were discussed. A collection of articles covering this aspect of 3D EM can also be found at https://biocomp.cnb.csic.es/3DEM-Methods/index.php/Main_Page.

## Conflict of Interest Statement

None declared.
